# Association Between Soluble Cell Adhesion Molecules (sP-Selectin, sE-Selectin, and sICAM-1) and Antibodies Against the Antigens of Proteus mirabilis in Rheumatoid Arthritis Patients

**DOI:** 10.7759/cureus.64942

**Published:** 2024-07-19

**Authors:** Georgios Christopoulos, Vasiliki Christopoulou, Konstantinos Stamatiou, Andreas Babionitakis, John G Routsias

**Affiliations:** 1 Department of Internal Medicine, Tzaneio General Hospital of Piraeus, Piraeus, GRC; 2 Department of Internal Medicine – Propaedeutic, Attikon University Hospital, Athens, GRC; 3 Department of Urology, Tzaneio General Hospital of Piraeus, Piraeus, GRC; 4 Department of Pathophysiology, Medical School National & Kapodistrian University of Athens, Athens, GRC; 5 Department of Microbiology, Medical School National & Kapodistrian University of Athens, Athens, GRC

**Keywords:** sp-selectin, se-selectin, rheumatoid arthritis, proteus mirabilis, sicam-1, adhesion molecules

## Abstract

Objective

The purpose of this study was to examine the association between the serum concentration of soluble cell adhesion molecules (CAMs) and antibodies against antigens of* Proteus mirabilis* (*P. mirabilis*) in rheumatoid arthritis (RA) patients, taking into consideration the implication of *P. mirabilis* in the etiopathogenesis of RA.

Methods

The serum levels of soluble P-selectin (sP-selectin), soluble E-selectin (sE-selectin), and soluble intercellular adhesion molecule-1 (sICAM-1) were determined by sandwich enzyme-linked immunosorbent assay (ELISA) in 59 RA patients and 36 healthy controls. Using the same ELISA method, the serum levels of class-specific antibodies against hemolysin (HpmB), urease C (UreC), and urease F (UreF) enzymes of *P. mirabilis* were also measured.

Results

In this study, increased levels of sP-selectin and sICAM-1 were observed in RA patients, while the levels of sE-selectin were increased in comparison with healthy controls but did not present a statistically significant difference. Moreover, increased levels of antibodies against HpmB, UreC, and UreF of *P. mirabilis* were found. Additionally, it was observed that the sE-selectin levels presented a significant correlation with IgG antibodies against the UreF antigen (there is no corresponding antigen in human tissue) in all the RA patients. A statistically significant correlation was observed between levels of soluble CAMs and antibodies against *P. mirabilis* in the different subgroups.

Conclusion

The observed correlation between soluble CAMs and antibodies against antigens of *P. mirabilis*, specifically in the subgroup of biologic therapy, indicates that *P. mirabilis* exists and provokes refractory in the treatment of RA.

## Introduction

Rheumatoid arthritis (RA) is an autoimmune disease characterized by chronic inflammation of unknown etiology. It affects approximately 1% of the general population [1,2]. Manifestations of systemic inflammation are common in RΑ; however, chronic inflammation of the synovial tissues is the most prominent symptom [3]. 

The pathogenic mechanisms of this disease are not fully understood; nevertheless, it is known that a combination of synovial tissue destruction processes, which involve lymphocytes, macrophages, and fibroblasts, with increased angiogenesis rates and proliferation of the synovium lining layer characterizes the initiation of RA development [4-7]. The complex cascade of inflammatory interactions characterizing RA is mediated by cell adhesion molecules (CAMs), such as P-selectin, E-selectin, and intercellular adhesion molecule-1 (ICAM-1) [8-10]. These glycoproteins mediate cell-cell and cell-extracellular matrix adhesion, which appears to play an important role in RA initiation and the perpetuation of synovial tissue inflammation [8].

More precisely, P-selectin, which is normally stored in the Weibel-Palade bodies of endothelial cells and in the alpha granules of platelets [11, 12], is released to the cell surface in response to the presence of autoantibodies and to inflammatory conditions in general [13, 14]. Released P-selectin produces CD40 ligand (sCD40L), which acts as a trigger for the activation of the endothelium [15, 16]. While P-selectin-induced endothelium activation results in a regular increase in vascular permeability, a synchronous motivation of activated platelets occurs. This leads to the migration of inflammatory agents to the joints. Indeed, low-affinity immunoglobulin (Ig) G receptors have been found to mediate platelet activation in the presence of anti-citrullinated protein antibodies [17].

Moreover, increased levels of soluble P-selectin (sP-selectin) and sCD40L found in the serum of RA patients correlated with RA severity [18, 19]. Using a mouse model of RA, Wipke et al. showed that after a few minutes of intravenous injection of autoantibodies, these antibodies migrated specifically to the joints even in the absence of pre-existing joint inflammation [20]. The specific homing of autoantibodies to the joints may contribute to the local activation of platelets, which in turn release important mediators of vascular permeability [21,22].

E-selectin is a cell adhesion molecule that is expressed on endothelial cells after activation by inflammatory cytokines or endotoxin [23,24]. Cell surface E-selectin contributes to the rolling attachment of leucocytes to the endothelium, a necessary step for leukocyte emigration into inflamed tissues, and has a main role in localized inflammatory responses [25, 26]. Soluble E-selectin (sE-selectin) is released from endothelial cells by proteolytic cleavage of the surface-expressed molecule [25,27].

Two studies found higher levels of sE-selectin in RA patients compared with healthy controls and osteoarthritis patients, respectively [28, 29]. In addition, a strong correlation between serum and synovial fluid concentrations of sE-selectin exists in RA patients [30]. Furthermore, synovial fluid levels of sE-selectin are higher in RA than in non-inflammatory joint diseases [31].

Inflammatory cytokines induce up-regulation of ICAM-1 expression on vascular endothelial cells, contributing to the adhesion of leucocytes to the local endothelial and subsequently to the migration of leukocytes to inflamed tissues [32,33]. Several authors demonstrated high soluble ICAM-1 (sICAM-1) levels in the serum of RA patients [28, 29], while others found that the increase in the serum of sICAM-1 in RA patients was not statistically significant [34,35].

Moreover, RA patients from various ethnic populations were found to have significantly elevated levels of antibodies against various antigens of *Proteus mirabilis* (*P. mirabilis*) [36]. In particular, it was observed that RA patients present asymptomatic bacteriuria more frequently in comparison to healthy controls. In addition, in the same study, it was observed that RA patients show increased levels of antibodies against *P. mirabilis* in their serum in comparison to healthy controls [37]. Similarly, in another study, it was found that RA patients present significantly increased levels of antibodies against *P. mirabilis* but not against *Escherichia coli* (*E. coli*) in comparison to healthy controls [38]. We found elevated levels of antibodies against hemolysin (HpmB), urease C (UreC), and urease F (UreF) enzymes of *P. mirabilis* in Greek RA patients [39]. In this study, we examined the existence of a correlation between the soluble cell adhesion molecules sP-selectin, sE-selectin, and sICAM-1 and the antibodies against HpmB, UreC, and UreF enzymes of *P. mirabilis* in RA patients.

## Materials and methods

This study included 59 RA patients (13 male and 46 female) with a mean age of 63.6 years and 36 healthy controls (10 male and 26 female) with a mean age of 60.9 years. The general characteristics of the participants are shown in Table 1. All patients included in this study had positive serum rheumatoid factor (RF) and fulfilled the diagnostic criteria for RA [40]. These were RA patients who attended the outpatient clinic of the rheumatology department. Patients with symptoms of urinary tract infections or patients who recently received antibiotic treatment were excluded from the study. Informed consent was obtained from all subjects.

**Table 1:** General characteristics of rheumatoid arthritis (RA) patients and healthy controls. The RA patients were subdivided into groups based on their received treatment. SD: standard deviation; ESR: erythrocyte sedimentation rate; CRP: C-reactive protein; N/A: not applicable; bDMARDs: biologic disease-modifying anti-rheumatic drugs

	RA patients	Healthy controls
Ν	59	36
Age (years) mean (SD)	63.6 (10.57)	60.9 (9.08)
Male/Female	13/46	10/26
Disease duration (years) mean (SD)	8.49 (6.23)	N/A
ESR (mm/hr) mean (SD)	55 (29.12)	Normal
CRP (mg/dl) mean (SD)	34.3 (49.31)	Normal
Without treatment	23	N/A
Combined	17	
Biologic therapy (bDMARDs)	19	

The erythrocyte sedimentation rate (ESR), serum C-reactive protein (CRP), RF, total blood count, and platelet (PLT) count were determined by routine methods. The participants were divided into three groups according to the treatment they received. The patients in the first group did not receive any treatment, while those in the second group received a combined treatment of conventional synthetic disease-modifying anti-rheumatic drugs (csDMARDs) with cortisone. The third group received biologic treatment.

sP-selectin, sE-selectin, and sICAM-1 were quantified in all patients and controls using Quantikine commercial sandwich immunoassays (R&D Systems, Minneapolis, MN) according to the manufacturer’s instructions. Briefly, a monoclonal antibody specific to the analyte was pre-coated onto a microplate. Standards, samples, and controls were pipetted into the wells. The dilution of the samples was 1/20 for sICAM-1 or sP-selectin assays and 1/10 for sE-selectin. A polyclonal antibody specific to the analyte, which had been conjugated to horseradish peroxidase, was used for detection. Following a wash to remove any unbound conjugated antibody, a substrate was added and color was developed, which was proportional to the analyte concentration. The intensity of the color was measured at 450 nm. The concentration of each sample was calculated from the standard curve and multiplied by the dilution factor.

The presence of antibodies against *P. mirabilis* in RA patients and healthy controls was determined by using enzyme-linked immunosorbent assays (ELISA) and three synthetic amino acid peptides that contained a homologous amino acid sequence with the *P. mirabilis* enzymes. Specifically, we used: (1) the peptide H2N-NGSSESRRALQDSQR-OH, which presents a homologous sequence with the HpmB of *P. mirabilis *and cross-reactivity with the human tissue antigen and specifically with the amino acid sequence EQK/RRAA (69-74) of the HLA-DRB1 molecule; (2) the peptide H2N-FAESRIRRETIAAED-OH, which presents a homologous sequence with the UreC of *P. mirabilis* and cross-reactivity with the human tissue antigen and specifically with the amino acid sequence LRREI (421-425) of α2(XI) collagens; and (3) the peptide H2N-ASRETKELRQEERQP-amide, which presents a homologous sequence with the UreF of *P. mirabilis* but not cross-reactivity with human tissue antigens.

For statistical analysis, the Student's t-test was used to compare the mean values of the soluble adhesion molecule levels between RA patients and healthy control subjects. The same statistical test was also used to compare the mean values of the antibodies against *P. mirabilis* antigens between the same groups. 

Similarly, a t-test was conducted to verify the age matching between patients and healthy controls, yielding t: 1.260 and p: 0.211. Since p >0.05, there was no significant difference in age between the two groups. Consequently, a chi-square test was performed to confirm gender matching, resulting in χ²: 1.577, p: 0.209. Similarly, the p >0.05 indicated no significant difference in gender distribution between patients and healthy controls. Thus, patients and healthy controls were well-matched in terms of both age and gender.

The samples were checked using D’Agostino-Pearson, Shapiro-Wilk, and Kolmogorov-Smirnov normality tests with GraphPad Prism version 9 (GraphPad Software, La Jolla, CA). These tests confirmed that the samples followed a normal (Gaussian) distribution. Therefore, a parametric analysis was performed.

Pearson’s correlation coefficient (r) was used for the detection of the relationship between the soluble adhesion molecules and antibodies against antigens of *P. mirabilis*, as well as ESR, CRP, and RF in the group and in all the subgroups of RA patients.

The local ethics committee of Laiko University Hospital approved the research protocol (approval number: 4837/21). Written and verbal informed consent was obtained prior to the study. All the procedures adhered to the ethical guidelines of the Declaration of Helsinki and its amendments. 

## Results

**Figure 1:**
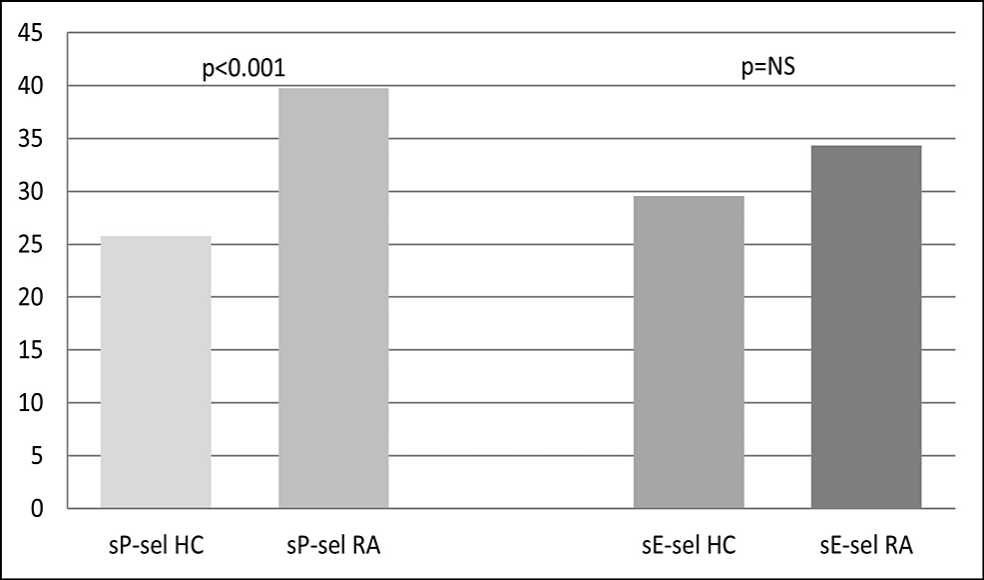
Serum concentrations of sP-selectin (sP-sel) and sE-selectin (sE-sel) in rheumatoid arthritis (RA) patients and healthy controls (HC) sP-selectin: soluble P-selectin;  sE-selectin: soluble E-selectin; NS: not significant

**Figure 2:**
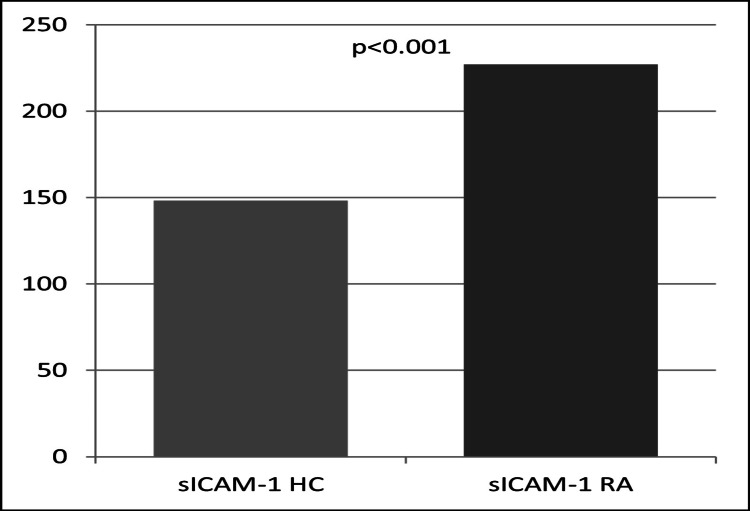
Serum concentrations of sICAM-1 in rheumatoid arthritis (RA) patients and healthy controls (HC) sICAM-1: soluble intercellular adhesion molecule-1

The comparison of the serum concentration of soluble CAMs between the RA patients and controls revealed differences between the two groups. The results of the serum concentration of soluble CAMs measured in the RA patients and controls are shown in Table 2. Specifically, the levels of sP-selectin were higher in the RA patients compared to those of healthy controls. The difference in sP-selectin levels between the two groups was statistically significant (t-test 6.023, p <0.001) (Figure 1). Similarly, the levels of sE-selectin were elevated in the RA patients but remained normal in the healthy controls. However, this difference was not statistically significant (p = NS) (Figure 1). The levels of sICAM-1 were also higher in the RA patients than in the normal controls. The difference in sICAM-1 levels between the two groups was statistically significant (t-test 6.496, p <0.001) (Figure 2).

**Table 2:** Results of the serum concentration of soluble adhesion molecules in RA patients and healthy controls Mean value (standard deviation) of sP-selectin, sE-selectin, and sICAM-1  in rheumatoid arthritis (RA) patients and healthy controls and the results of statistical analysis t-test sP-selectin: soluble P-selectin;  sE-selectin: soluble E-selectin; sICAM-1: soluble intercellular adhesion molecule-1

	RA patients	Healthy controls	t-Test
	N=59	N=36	p-value
sP-selectin (ng/ml) Mean (SD)	39.74 (15.08)	25.79 (7.36)	p<0.001
sE-selectin (ng/ml) Mean (SD)	34.33 (17.01)	29.57 (10.70)	p = NS
sICAM-1 (ng/ml) Mean (SD)	227.10 (76.96)	148.41 (40.83)	p<0.001

Regarding antibodies against the antigens of *P. mirabilis* enzymes, their levels in the RA patients were significantly increased compared with the healthy control subjects (Table 3). In particular, the RA patients showed statistically significant raised levels of antibodies against HpmB compared with the healthy control subjects (Table 3). Likewise, the RA patients showed significantly higher levels of antibodies against UreC compared with the healthy control subjects (Table 3).

**Table 3:** Results of the antibodies against antigens of Proteus mirabilis in rheumatoid arthritis (RA) patients and healthy controls Mean values (MV±SD, OD 405) of the different classes of antibodies against antigens of *P. mirabilis* in RA patients and healthy controls, and the results of the statistical analysis t-test. HpmB: hemolysin; UreC: urease C; UreF: urease F

		RA patients	Healthy controls	t-test
		N=59	N=36	p-value
Anti-HpmB	lgM	0.91±0.61	0.66±0.31	p=0.008
	IgG	1.11±0.59	0.69±0.20	p<0.001
	IgA	0.37±0.21	0.22±0.07	p<0.001
Anti-UreC	lgM	0.62±0.39	0.45±0.23	p<0.01
	IgG	0.99±0.72	0.65±0.22	p=0.001
	IgA	0.26±0.11	0.19±0.05	p<0.001
Anti-UreF	lgM	0.95±0.74	0.65±0.37	p<0.01
	IgG	0.93±0.50	0.63±0.19	p<0.001
	IgA	0.30±0.12	0.21±0.06	p<0.001

Moreover, the RA patients showed significantly raised levels of antibodies against the UreF antigen; there is no corresponding antigen in the tissues of the human body. Similarly, the differences in antibody levels against UreF between the RA patients and the healthy control subjects were statistically significant. (Table 3).

In this study, the levels of sP-selectin, sE-selectin, and sICAM-1 were associated with the levels of antibodies against the three antigens of *P. mirabilis*: HpmB, UreC, and UreF. Our findings revealed that the sP-selectin levels in all the RA patients did not present a significant correlation. However, in the subgroup of RA patients without treatment, a significant correlation was observed between sP-selectin levels and IgM (r = 0.661, p <0.004) and IgG antibodies (r = 0.574, p <0.01) against HpmB. In the subgroup of RA patients treated with biologic therapy, an important correlation was also observed between sP-selectin levels and IgM antibodies against HpmB (r = 0.469, p <0.04). The concentration of sE-selectin in all the RA patients showed a strong correlation with IgG antibodies against UreF (r = 0.301, p <0.02). Equally strong was the correlation between the levels of sE-selectin and the IgA antibodies against HpmB in the RA patients treated with biologic therapy (r = 0.515, p <0.02).

While the sICAM-1 levels in all the RA patients did not present a significant correlation with the levels of antibodies against antigens of *P. mirabilis*, in the subgroup of RA patients treated with biologic agents, a statistically significant correlation was shown between sICAM-1 concentration and IgM antibodies against HpmB (r = 0.506, p<0.02). Moreover, a statistically significant correlation was found between sICAM-1 levels and CRP levels (r = 0.317, p<0.01) in all the RA patients. Also, a statistically significant correlation was found between sICAM-1 and sP-selectin levels (r = 0.232, p<0.02) in all the RA patients.

## Discussion

Soluble forms of P-selectin, E-selectin, and ΙCAM-1 are known to exist in human serum and present elevated levels in numerous diseases. Previous studies have demonstrated that in RA, the levels of circulating sICAM-1 and sP-selectin were significantly elevated in RA patients relative to healthy controls [28]. Likewise, in this study, the levels of sP-selectin and sICAM-1 were significantly elevated in RA patients in comparison with healthy controls. While the levels of sE-selectin were increased in RA patients in comparison with healthy controls, this difference was not statistically significant. This result was in accordance with that of previous studies, which showed that the levels of sE-selectin were raised in RA patients compared with healthy controls, even though this difference was not statistically significant [28, 41].

Previous studies have shown that RA patients have elevated antibodies against various antigens of *P. mirabilis* [36, 42]. We demonstrated that the levels of sP-selectin, sE-selectin, and sICAM-1 in RA patients correlate with the levels of antibodies against HpmB, UreC, and UreF of *P. mirabilis*, further supporting the implication of *P. mirabilis* in the etiopathogenesis of RA [39]. In this study, we found increased levels of sP-selectin; this result is in accordance with previous studies [17,18,28]. Our finding of a statistically significant correlation of sP-selectin levels with IgM and IgG antibodies against HpmB in the subgroup of RA patients without treatment suggests that the antibodies against *P. mirabilis* through molecular mimicry and cross-reaction mechanisms with tissue antigens of the joints provoke an increased expression of sP-selectin, which contributes to the inflammation of the joints and the activation of RA.

Likewise, the significant correlation of sP-selectin with IgM antibodies against HpmB in the subgroup of biologic therapy shows disease activity despite treatment. This finding may indicate continuous local triggering by *P. mirabilis* and a rapid endothelial cell response consisting of the release of P-selectin [43, 44]. Indeed, it has been shown that the expression of P-selectin on the cell surface occurs within one minute after endothelial activation [14]. Moreover, the increased levels of sP-selectin found in the synovial liquid of RA patients are mainly released by synovial microvascular endothelial cells [28,45,46]. By contrast, decreased levels of sP-selectin in RA patients have been observed after infliximab infusions [47]. As shown in experimental models of inflammation, the blocking of P-selectin provokes a significant decrease in leukocyte rolling [48]. In our study, sP-selectin levels did not present a correlation with the antibodies against *P. mirabilis* in all the RA patients, obviously because most of them were receiving treatment, resulting in decreased expression of sP-selectin and also decreased production of antibodies against *P. mirabilis*.

Similarly, previous studies found that RA patients with active disease have higher levels of sE-selectin; however, after treatment with biologic agents or methotrexate, the levels of sE-selectin gradually decrease [49-51]. In our study, the significant correlation of sE-selectin levels with the IgA antibodies against HpmB in the subgroup of biologic therapy showed that sE-selectin collaborates with the antibodies in the development of inflammation in the joints. In addition, non-response to treatment with biological agents is probably due to the abovementioned triggering of joint inflammation by *Proteus*. This fact is strengthened by the significant correlation of sE-selectin with IgG antibodies against the UreF antigen of *P. mirabilis* in all the RA patients, whereas there is no corresponding antigen in the tissues of the human body. Consequently, the finding of these antibodies should be attributed to the presence of *P. mirabilis* in RA patients [29].

Furthermore, this study revealed a statistically significant difference in the levels of sICAM-1 between RA patients and healthy controls. This finding is in accordance with those of previous studies [28,50,52]. However, other investigators have failed to demonstrate significant differences in sICAM-1 levels between RA patients and healthy controls [34,35,53].

Also in our study, sICAM-1 levels were significantly correlated with CRP levels. This result was similar to that of previous studies [52,54]. Notably, high circulating levels of sICAM-1 have been observed in RA patients with active disease, suggesting sICAM-1 levels as a marker for endothelial activation [55-57]. In confirmation of the above, a significant correlation was demonstrated between the serum levels of sICAM-1 and sP-selectin in this study. This correlation shows disease activity because sP-selectin is associated with disease activity in RA patients [17,18,28].

According to several researchers, RA patients under treatment have decreased levels of sICAM-1 [47,51,58]. This fact was also present in our study. In addition to decreased levels of sICAM-1, the patients under treatment also had decreased levels of antibodies against *P. mirabilis*. In the subgroup of RA patients who received biologic therapy, a significant correlation was observed between sICAM-1 level and IgM antibodies against HpmB, which suggests the triggering of RA by *P. mirabilis*. The consequent production of antibodies against *P. mirabilis* contributes to the expression of ICAM-1 in synovial tissues and the development of joint inflammation.

Antibodies against *P. mirabilis* were found in the serum of RA patients even in the initial stages of the disease. [59]. This bacterium can potentially be implicated in the induction of RA via molecular mimicry between bacterial and self-antigens that may lead to the production of antibodies targeting both bacteria and self-tissues [60]. Prior research has also shown that anti-*Proteus *antibiotics in combination with anti-rheumatic drugs in RA patients seem to have a better response to disease activity [61-63]. Finally, other anti-*Proteus *measures, in the form of vegetarian diets and phytotherapy, appear to have beneficial effects for RA patients [64-66].

The limitations of this study are a relatively small sample size. Further studies are required, with follow-up after antibiotic treatment in RA patients who present increased levels of antibodies against *P. mirabilis*. 

## Conclusions

Soluble CAMs are involved in the process of leucocyte adhesion and ingress into the rheumatoid synovium, causing the initiation and progression of the disease. Increased levels of antibodies against HpmB, UreC, and UreF of *P. mirabilis* and increased levels of sP-selectin, sE-selectin, and sΙCAM-1 were observed in this study. Moreover, the observed correlation between these antibodies and soluble CAMs suggests that *P. mirabilis* triggers the production of these antibodies in RA patients, which, through molecular mimicry and cross-reaction mechanisms with self-antigens of the joints, provokes activation of RA.
